# Regarding the significance of plasmatic arginine/homoarginine in neurogenic pulmonary edema following surgery of medulla oblongata tumors patients

**DOI:** 10.1097/JS9.0000000000001085

**Published:** 2024-01-18

**Authors:** Wei Yang, Jinlin Liu

**Affiliations:** a Department of Clinical Laboratory, Zhejiang Provincial People’s Hospital Bijie Hospital, Bijie, Guizhou; b Department of Clinical Laboratory, South China Hospital, Medical School, Shenzhen University, Shenzhen, People’s Republic of China

*Dear Editor*,

We read Wang’s study^1^ with great interest. In this prospective clinical study, the author aims to investigate the fluctuations of neurotransmitters in peripheral venous blood during the perioperative period and to identify independent predictors for postoperative neurogenic pulmonary edema (NPE) in patients with medulla oblongata-involved tumors. They found that decreased concentrations of arginine and homoarginine, the absence of intraoperative use of sevoflurane, and prolonged postoperative tracheal extubation were independent risk factors for postoperative NPE in these 36 medulla oblongata-involved tumor patients. Then, they concluded that these two neurotransmitters’ concentrations could serve as potential early warning indicators of postoperative NPE in clinical practice. Despite definite results, we raise some concerns and comments on this study.

First, these neurotransmitters’ concentrations in a neurosurgical skull base tumor patients’ group at seven perioperative time points are lacking in Wang’s study. Thus, it is challenging to pinpoint the definite association because there is little information on 39 neurotransmitters in other neurosurgical skull base tumor patients. Without appropriate controls, these two neurotransmitters could be a bystander effect in these neurosurgical skull base tumor patients.

Second, the sample size of univariate and multivariate Cox proportional hazards is usually more than 15 times the confounder. The more confounders are analyzed, the more sample sizes are required. In Wang’s study, 17 confounders including male, age, without a history of hypertension, medulla oblongata originated *et al.*, and 39 other neurotransmitters confounders were all included. However, the sample size was only 36 patients with medulla oblongata-involved tumors. Thus, the statistical significance of univariate or multivariate analysis of risk factors could result in inappropriate results in Khalid’s study.

Finally, in this letter, we want to discuss whether a clear association exists between the warning indicators of arginine and homoarginine with postoperative NPE in clinical practice. However, although we felt the need to address this point, we congratulate Zhao’s outstanding work^1^, which gives us this opportunity to discuss the significance of plasmatic arginine/homoarginine in NPE following surgery of medulla oblongata tumor patients.

## Ethical approval

Not applicable.

## Consent

Not applicable.

## Sources of funding

Not applicable.

## Author contribution

W.Y. and J.L.: discussed and wrote the manuscript.

## Conflicts of interest disclosure

We declare no conflicts of interest.

## Research registration unique identifying number (UIN)

Not applicable.

## Guarantor

Jinlin Liu.

## Data availability statement

Not applicable.

## Provenance and peer review

Not applicable.

## Data availability statement

Not applicable.
